# The role of daughters in relation to their mother’s cervical cancer diagnosis and treatment in Guatemala: a descriptive study

**DOI:** 10.1186/s12905-023-02305-3

**Published:** 2023-03-29

**Authors:** Hallie Dau, Anna Gottschlich, Lynn Metz, Natalia Pineda, Andres Pineda, Christian S. Alvarez, Kristin Bevliacqua, Carlos Mendoza-Montano, Gina Ogilvie, Alvaro Rivera-Andrade, Eduardo Gharzouzi, Rafael Meza

**Affiliations:** 1 School of Population and Public Health, The University of British Columbia, Vancouver, BC Canada; 2 Women’s Health Research Institute, BC Women’s Hospital and Health Centre, Room H203J - 4500 Oak Street, Vancouver, BC V6H 3N1 Canada; 3 School of Public Health, University of Michigan, Ann Arbor, MI USA; 4 Institute of Nutrition of Central America and Panama, Research Center for the Prevention of Chronic Diseases, Guatemala City, Guatemala; 5 Department of Population, Family and Reproductive Health, Johns Hopkins Bloomberg School of Public Health, Baltimore, MD USA; 6 Instituto Nacional de Cancerología, Guatemala City, Guatemala

**Keywords:** Guatemala, Cervical cancer, Daughter, Support, Caregiver

## Abstract

**Purpose:**

There is currently no information on how caregivers for women diagnosed with cervical cancer in Guatemala, particularly daughters, are affected by their supportive role. This study’s objective was to describe the support role of caregivers in the country, with a focus on daughters with a mother diagnosed with cervical cancer.

**Methods:**

This analysis utilizes data from a cross-sectional study which aimed to understand pathways to cervical cancer care. Women seeking cervical cancer treatment at the Instituto de Cancerologia (INCAN) in Guatemala City, Guatemala and their companions were surveyed. Descriptive statistics were calculated.

**Results:**

One hundred forty-five women seeking treatment and 71 companions participated in the study. Patient's daughters were most frequently reported as the person who provided the most support (51%) and as the most reported to have encouraged the patient to seek care. Furthermore, daughters were noted as the person most reported to fulfill the major household and livelihood roles of the patient while they were seeking or receiving treatment (38.0%). Most daughters reported that they were missing housework (77%), childcare (63%), and income-earning activities (60%) to attend the appointment with their mothers.

**Conclusion:**

Our study suggests that in Guatemala cervical cancer patient's daughters have a significant support role in their mother’s cancer diagnosis. Furthermore, we found that while caring for their mothers, daughters in Guatemala are often unable to participate in their primary labor activities. This highlights the additional burden that cervical cancer has on women in Latin America.

## Introduction

Cervical cancer is largely preventable through screening and Human Papillomavirus (HPV) vaccination [1]. However, due to a lack of widespread comprehensive screening programs as well as historically underfunded healthcare systems, [2, 3] it remains the second most common cancer for women living in low- and middle-income countries (LMIC) [4]. Among women in LMICs who do seek care, many face a limited number of publicly available centers that provide effective screening and cancer care, as well as lacking availability of trained medical staff, leaving many women unscreened or unable to complete treatment for premalignant lesions or cancers [5–7]. As a result, women in LMICs are more likely to be diagnosed with late stages of the disease [8] and 88% of global cervical cancer deaths occur in these settings [4].

As in other LMIC settings, the burden of cervical cancer in Guatemala is high. The adjusted standard mortality rate of cervical cancer in Guatemala is 11.9 deaths per 100,000 person-years, which is six times higher than the United States (2.1 deaths per 1000,000 person years) [9]. At the country level there are only a handful of specialized public hospitals providing services to cancer patients. Thus, most patients are treated at tertiary care hospitals located in Guatemala City, requiring many women to travel from across the country to seek cervical cancer treatment. Given that the capacity and resources of these public hospitals are limited, patients are often referred to the Instituto Nacional de Cancerología (INCAN), the only non-profit hospital in Guatemala specializing in cancer treatment for adults, usually at no cost. However, women can attend INCAN directly and pay small fees to get attention if they wish so. Women at INCAN have access to chemotherapy, radiation, and surgery. The type of treatment and its length depends on the stage at diagnosis and treatment availability at that time. As a result, the lack of widespread services has led to unsurprisingly inconsistent care and treatment. A 2019 study by Zamorano et al. reviewed the charts of women seeking care at INCAN from 2005–2007 and found that approximately 25% of women diagnosed did not initiate care after their diagnosis and a further 20% were lost to follow up after six months [10].

Existent research has focused on the role of spouses [11–13] and children in general [14, 15] with regards to a woman’s cervical cancer diagnosis in LMICs. However, there is limited knowledge on the support role of other caregivers, and more specifically on the role of patient’s daughters in relation to their mother’s cervical cancer diagnosis. To our knowledge this is the first study to focus on the support role of daughters with mothers diagnosed with cervical cancer in Guatemala. The gendered nature of caregiving necessitates an improved understanding of the support provided by daughters, as women in these countries typically bear the burden of the majority of paid and unpaid caring work [16–18]. This in turn limits their ability to access educational and employment opportunities. The present study aims to describe which individuals provide the most support to women seeking cervical cancer care in Guatemala and assess the burden of care for the daughters of women seeking cervical cancer care.

## Methods

### Study population

This study utilizes data from a cross-sectional study conducted at INCAN that aimed to understand the pathways to care for cervical cancer. The study recruited women seeking cervical cancer care and their companions. Women were eligible for the study if they were (**i**) currently seeking cervical cancer treatment at INCAN; (**ii**) over the age of 18; and (**iii**) spoke Spanish or were accompanied by someone who could act as a translator. Companions were eligible for the study if they (**i**) were accompanying an individual with cervical cancer seeking treatment at INCAN who had consented to participate in the study; (**ii**) over the age of 18; and (**iii**) spoke Spanish.

### Data collection

Women were recruited using convenience sampling from July 24th to September 1^st^, 2017. Participants were recruited from the outpatient clinic for gynecologic and breast cancers, the inpatient and outpatient radiotherapy departments, and the outpatient chemotherapy department. Participants received 40 Quetzales (approximately 5.20 USD) as compensation for their time participating in the study.

Two trained interviewers fluent in Spanish administered surveys on tablets using the Qualtrics offline application. Patients and their companions were administered surveys separately and surveys were tailored to the participant (i.e., patient or companion-specific survey). Questions were read aloud to participants and entered into Qualtrics by study personnel. All answers were self-reported.

### Measures and analysis

#### Patient survey

The patient survey consisted of 113 questions that took approximately 30 to 60 min to complete. The survey included questions on the participant’s demographics, access to healthcare, cervical cancer screening and treatment history, knowledge of cervical cancer and HPV, barriers to seeking treatment, and the patient’s social support network. The survey utilized validated questions from a 2017 survey in Guatemala developed Gottschlich et al. [19] as well as the breast module of the Cancer Awareness Measure, adapted to the Guatemalan cervical cancer population [20]. The tool was piloted with a group of volunteers.

Primary outcomes for the patient survey included relationship with main source of support, with whom patient first disclosed diagnosis, and relationship with individual who encouraged patient to see a doctor were assessed. These questions had the response options daughter, spouse/partner, son, mother, father, sister, brother, aunt, uncle, male friend, female friend, a member of their religious community, and other. Due to small cell sizes, all responses except for spouse/partner, mother, son, and daughter were recategorized as “other”. Daughter-in-laws were categorized as daughter in both the patient and companion survey data.

Data related to questions regarding to the demographics and cancer characteristics of women seeking cervical cancer care such as age, ethnicity, native language, education, literacy, income, marital status, number of children, travel time to appointment, cancer stage, time since diagnosis, time between symptoms and seeking care, and sought out screening because felt sick were also summarized. To confirm cancer stage, we extracted all available medical charts for participants.

#### Companion survey

The companion survey consisted of 52 questions and took approximately 15 min to complete. The survey included questions on the companion’s demographics, relationship to the patient, and the impact of cervical cancer treatment on the patient and family, including the companion.

Primary outcomes were what activities the companion missed to attend the appointment (income earning activities, caring for an elderly relative, caring for children, housework, social activities, religious activities, school), number of previous visits by companion to INCAN, and amount of time typically spent at INCAN by companion. Companions also reported who fulfils the major roles of the patients when she is seeking treatment, and which individuals were most impacted by the woman’s cervical cancer diagnosis (daughter, spouse/partner, son, mother, father, sister, brother, aunt, uncle, male friend, female friend, and other).

Data related to questions from the companion’s survey including language, relationship, distance travelled from outside Guatemala City, anticipated number of days attending treatment appointments with your companion, accommodation, and transportation were also summarized.

#### Analysis

All data was analyzed using R 4.0.5 [21]. Descriptive statistics were calculated using counts and frequencies. After investigating the primary outcomes from the companion survey, the dataset was subset to only include daughters and the analyses were rerun.

### Ethics

Ethical approval was obtained from the ethics committee of University of Michigan (IRB# HUM00127150, INCAN, and the Institute of Nutrition of Central America and Panama (CIE-REV 069/2017). All study participants provided written informed consent.

## Results

### Cervical cancer patients

Of the 145 women diagnosed with cervical cancer who completed the survey, most identified as Ladina (non-indigenous) (77.9%), had less than a primary school education (89.0%), were married (58.9%), and had more than five children (42.7%) (Table 1). Additionally, the majority of the women self-reported that they were diagnosed with Stage II or III cervical cancer and over 85% of women sought out treatment because they were experiencing symptoms. Of the 145 participants, 112 had medical records available for abstraction. From these records, we saw that nearly 90% of the women with available medical records were actually diagnosed at later stages (IIB-IV).

**Table 1:** Demographic and cancer characteristics of women seeking cervical cancer care

Variable name	n (%)
Sample size	145 (100)
Age in years	
25–34	7 (4.8)
35–44	35 (24.1)
45–54	41 (28.3)
56–64	35 (24.1)
65–74	22 (15.2)
75 +	5 (3.4)
Ethnicity	
Ladina	113 (77.9)
Maya	30 (20.7)
Other	2 (1.4)
Native language	
Spanish	122 (84.1)
Other	23 (15.9)
Education	
Primary school or less	129 (89.0)
More than primary school	16 (11.0)
Literate (yes)	100 (69.0)
Income (Quetzales)	
0–500	56 (38.9)
501–1000	40 (27.8)
1001–2000	38 (26.4)
2001–3000	7 (4.8)
≥ 3001	4 (2.8)
Marital status	
Single	29 (20.0)
Married	76 (52.4)
Previously married	40 (27.6)
Number of children	
None	1 (0.7)
1–2	32 (22.1)
3–4	49 (33.8)
5 +	61 (42.1)
*Missing*	2 (1.4)
Travel time to INCAN	
< 1 h	22 (15.2)
1—4 h	81 (55.9)
5—12 h	35 (24.1)
> 12 h	5 (3.4)
Do not know	2 (1.4)
Self-reported stage at diagnosis	
Stage 1	4 (2.8)
Stage 2	37 (25.5)
Stage 3	30 (20.7)
Stage 4	6 (4.1)
*Missing*	*68 (46.9)*
Time since diagnosis	
< 1 month	5 (3.4)
1–5 months	42 (29.0)
6–11 months	23 (15.9)
1- 3 years ago	47 (32.4)
3 + years ago	27 (18.7)
I don't know/I don't remember	1 (0.7)
Time between symptoms and seeking care	
< 1 month	52 (35.9)
1–5 months	54 (37.2)
6–11 months	10 (6.9)
More than 1 year	26 (17.9)
*Missing*	3 (2.1)
Sought out screening because felt sick (yes)	125 (86.2)

**Fig. 1:**
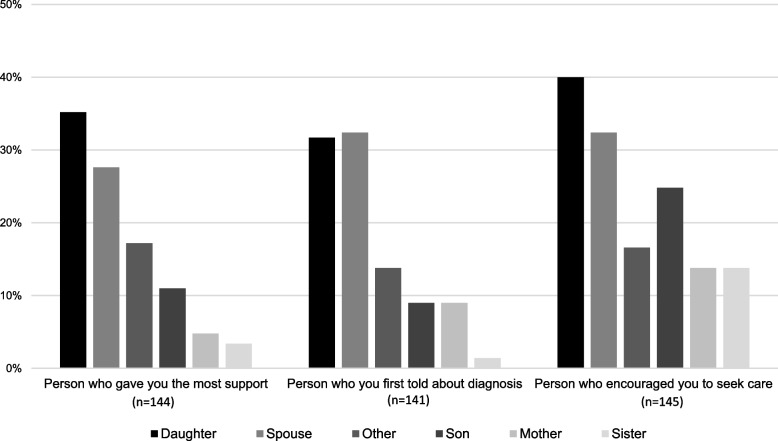
Type of support provided to women being treated for cervical cancer stratified by individual classification

Women seeking treatment for cervical cancer identified daughters as the person who provides the most overall social support (Fig. 1). Furthermore, daughters were reported as the person most reported to encourage them to seek care (40.0%). When asked about the first person to whom the participants disclosed their diagnosis, the spouse was the most common response (32.4%) followed closely by daughters (31.7%).

### Companions

In all, 71 companions completed the survey (Table 2). The majority of companions were under the age of 45 (64.8%). The majority were daughters of the women being treated for cervical cancer (49.3%), followed by their spouses (21.1%). About 75% of the companions reported traveling from outside Guatemala City and about 76% reported using the public bus to travel to INCAN.

**Table 2:** Characteristics of companions

Variable	n (%)
Sample size	71 (100)
Age in years	
18–24	12 (16.9)
25–34	14 (19.7)
35–44	20 (28.2)
45–54	17 (23.9)
56–64	8 (11.3)
Language	
Spanish	70 (98.6)
Kaqchikel	1 (1.4)
Relationship to patient	
Daughter	35 (49.3)
Spouse/Partner	15 (21.1)
Son	6 (8.5)
Mother	3 (4.2)
Other	7 (9.9)
*Missing*	*5 (7.0)*
Travelled from outside Guatemala City	
Yes	53 (74.6)
No	18 (25.4)
Anticipated number of days attending treatment appointments with your companion?	
< 1 day	15 (21.1)
2–3 days	17 (23.9)
4–7 days	10 (14.1)
8 + days	24 (33.8)
*Missing*	*5 (7.0)*
Where do you usually stay when you accompany your companion to INCAN?	
Friend/family	19 (26.8)
Hotel	11 (15.5)
My house	1 (1.4)
I don't have anywhere to stay	17 (23.9)
*Missing*	*23 (32.4)*
Transportation type to INCAN	
Public bus	54 (76.1)
Personal vehicle	6 (8.5)
Multiple forms of transportation	6 (8.5)
Taxi	3 (4.2)
Walk	1 (1.4)
Other	1 (1.4)

When asked what person was most impacted by the woman’s cervical cancer diagnosis, most companions reported that daughters were the most impacted (59.2%). This was followed by sons (53.5%) and sisters (45.1%). Fathers were identified as one of the least impacted according to the patient’s companions (7.0%).

### Daughter companions

**Fig. 2:**
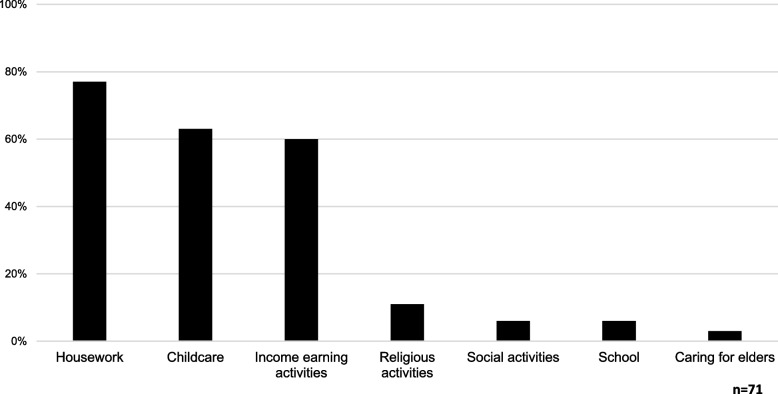
Reported activities missed by daughters serving as companions to their mother’s cervical cancer appointments

The companions reported that daughters were most reported to fulfill the major roles of the patient when they are seeking treatment (38.0%). Major roles included incoming earning, caring for children and elderly family members, and caring for home. Moreover, 37.1% of daughters who served as companions reported that they anticipated that they would be away from home for more than eight hours. Additionally, 80% of daughters reported that they have accompanied their mother for treatment three or more times and approximately 63% believed that they would need to accompany their mother for treatment at least three more times. The majority of companion daughters (77%) reported that they were missing housework/childcare (63%), and income earning activities (60%) (Fig. 2). Additionally, 5.7% of the daughter companions reported having to miss school to accompany their mother to treatment.

## Discussion

This study included 145 women attending cervical cancer treatment and 71 companions at INCAN in Guatemala City. Findings showed that daughters have a large role in the support of cervical cancer patients undergoing treatment at INCAN, as reported by both the patients and their companions.

The large support role of daughters in their mother’s cervical cancer diagnosis in Guatemala is unique because spouses have largely been reported to provide the most support for individuals diagnosed with cancer in other settings [22, 23]. Within current literature there is a limited understanding of the support role of daughters in relation to their mother’s cervical cancer diagnosis. Most of the current relevant research focuses on the relationship between women diagnosed with breast cancer and their daughters [24, 25]. There is a clear research gap in understanding the relationship among women diagnosed with cervical cancer and their daughters, particularly in LMICs. It is important to understand the daughter-caregiver relationship, not only to allow for the provision of additional support for women diagnosed with cervical cancer, but also for their caregiving daughters. A 1998 study by Raveis et al. of 164 cancer outpatients and their adult caregiving daughters found that caregiver burden was significantly correlated with depression [26]. As such, a reliance on daughters can have a negative psychological burden for those providing support, highlighting a double burden of cervical cancer in women in which in not only impacts women diagnosed, but their caregiver as well.

Cervical cancer can be a stigmatizing disease. Many women report embarrassment and shame due to the location of the cancer as well as the connection of the disease to HPV, a sexually transmitted infection [27–29]. As a result, many women report feelings of isolation [28], being a burden to others [29], and verbal abuse [27] after their diagnosis. Prior research has shown that a cervical cancer diagnosis can have a negative impact on the relationship a woman has with her partner and the support she receives, particularly in LMICs [11–13]. For example, Rosser et al. conducted a qualitative study among 110 men in Western Kenya about their knowledge and attitudes about cervical cancer. Only 21.8% of men reported that they would try and assist their partners if they were diagnosed with cervical cancer [30]. In fact, many women report spousal desertion after their cervical cancer diagnosis [31, 32]. Women in Guatemala may feel shame and fear in speaking about their diagnosis with their partners. Futheremore, there is also the issue of the “machista” culture, women are seen as inferior, and with a disease that affects their “womanness “ they are viewed as even less worth. Many male family members do not believe that it is their role to look after sick female members of their family. Rather boys are pulled out of school to work to meet household financial needs while girls are asked to care for the sick [33, 34]. With this, it is unsurprising that daughters play an important role in the support of their mother’s cervical cancer diagnosis. In addition to expanding screening programs, one way to ease the burden of support from daughters would be to provide additional education about cervical cancer to male caregivers and other uninformed relatives and friends to help reduce the stigmatization of the disease. Increased knowledge about HPV and education has been shown to reduce overall stigma of the disease [30, 35].

Prior research has shown that daughters are often expected to fulfill caregiver roles for ailing parents [36–39]. Our findings support this as daughters were shown to be the primary support for their mothers diagnosed with cervical cancer in this Guatemalan patient’s sample. In addition to attending multiple day long trips to the cancer clinic, daughters also noted that they missed labor-related tasks such as childcare and income earning activities. By placing the caregiving role of a woman’s cervical cancer diagnosis primarily on daughters, it prevents the caregiving daughters from consistently providing care to their own children, continuing their education or performing paid or unpaid labor. The latter is important because most of the unpaid labor is provided by women [40]. Similarly, previous research has demonstrated that a women’s cervical cancer diagnosis impacts her ability to maintain employment [28, 41, 42] and ability to care for her children [43]. As such, a cervical cancer diagnosis economically hinders both the women diagnosed as well as their daughters. This is important because women in Guatemala are already disadvantaged in terms of education, labor force participation, financial independence, and political representation [44].

This study has several strengths and limitations. First, this study is strengthened by its novelty, as to our knowledge, this is the first study to examine the relationship between cervical cancer patients and their daughters in Guatemala. Additionally, this study was conducted in the only specialized center providing public cancer care in Guatemala, which sees patients from all over the country. With regards to limitations, this study was limited to self-reported measures. Women were asked to self-report measures such as time since diagnosis and types of available health centers in their community, which could lead to recall bias. We were also limited in our understanding of the number of women who had daughters and daughter in laws as this information was not collected. Rather, we were only able to report on the number of women who brought their daughter or daughter-in-law to the clinic that day. Furthermore, non-probabilistic sampling methods were used to recruit women which could lead to selection bias in the study results. Similarly, as this study was conducted at one treatment center, it is likely not a representative sample of all women diagnosed with cervical cancer in Guatemala.

Our study indicates that women diagnosed with cervical cancer in Guatemala rely predominately on their daughters for support. There is a need to further explore the reliance on daughters for support from women diagnosed with cervical cancer not only in Guatemala but in other LMICs. Both quantitative and qualitative methods could provide a greater understanding of the role daughters have in providing support. Additionally, more research is needed on how to best support daughters while caring for their mothers and ensure that they are being screened for cervical cancer. Furthering this research will inform policymakers and stakeholders in Guatemala and Central America with information on how to support both women diagnosed with cervical cancer and their daughters, in order to promote gender equity—goal five of the sustainable development goals [45]. Furthermore, this evidence on the burden of cervical cancer on daughters in Guatemala provides global policymakers with additional evidence for expanding cervical cancer screening programs, which will help lead to the eventual elimination of cervical cancer worldwide.

In conclusion, our study suggests that in Guatemala cervical cancer patient's daughters have a significant support role in their mother’s cancer diagnosis. Furthermore, we found that while caring for their mothers, daughters in Guatemala are often unable to participate in their primary labor activities. This highlights the additional burden that cervical cancer has on women in Latin America.

## Data Availability

The datasets used and/or analyzed during the current study available from the corresponding author on reasonable request.

## References

[1] The Lancet (2020). Eliminating cervical cancer. Lancet.

[2] Anyangwe SC, Mtonga C (2007). Inequities in the global health workforce: the greatest impediment to health in sub-Saharan Africa. Int J Environ Res Public Health.

[3] Louie KS, De Sanjose S, Mayaud P (2009). Epidemiology and prevention of human papillomavirus and cervical cancer in sub-Saharan Africa: a comprehensive review. Tropical Med Int Health.

[4] Arbyn M, Weiderpass E, Bruni L, de Sanjosé S, Saraiya M, Ferlay J, Bray F (2020). Estimates of incidence and mortality of cervical cancer in 2018: a worldwide analysis. Lancet Glob Health.

[5] Johnston C, Ng JS, Manchanda R, Tsunoda AT, Chuang L (2017). Variations in gynecologic oncology training in low (LIC) and middle income (MIC) countries (LMICs): Common efforts and challenges. Gynecol Oncol Rep.

[6] Nakaganda A, Solt K, Kwagonza L, Driscoll D, Kampi R, Orem J (2021). Challenges faced by cancer patients in Uganda: Implications for health systems strengthening in resource limited settings. J Cancer Policy.

[7] Rakhshanda S, Dalal K, Chowdhury HA, Mayaboti CA, Paromita P, Rahman AKMF, Hussain AHME, Mashreky SR (2021). Assessing service availability and readiness to manage cervical cancer in Bangladesh. BMC Cancer.

[8] Vu M, Yu J, Awolude OA, Chuang L (2018). Cervical cancer worldwide. Curr Probl Cancer.

[9] Sung H, Ferlay J, Siegel RL, Laversanne M, Soerjomataram I, Jemal A, Bray F (2021). Global cancer statistics 2020: GLOBOCAN estimates of incidence and mortality worldwide for 36 cancers in 185 countries. CA Cancer J Clin.

[10] Zamorano AS, Barnoya J, Gharzouzi E, Chrisman Robbins C, Orozco E, Polo Guerra S, Mutch DG (2019). Treatment compliance as a major barrier to optimal cervical cancer treatment in Guatemala. J Glob Oncol.

[11] Amoo EO, Olawole-Isaac A, Okorie N, Ajayi MP, Adekola PO, Amana TR, Olu-Owolabi F (2018). Spousal desertion and coping strategies among women with cervical cancer in Nigeria: a schematic framework for wellbeing. Afr Popul Stud.

[12] Afiyanti Y, Milanti A (2013). Physical sexual and intimate relationship concerns among Indonesian cervical cancer survivors: A phenomenological study. Nurs Health Sci.

[13] Kebede W, Kebede K (2017). Psychosocial experiences and needs of women diagnosed with cervical cancer in Ethiopia. Int Soc Work.

[14] Kamau RK, Osoti AO, Njuguna EM (2007). Effect of diagnosis and treatment of inoperable cervical cancer on quality of life among women receiving radiotherapy at Kenyatta National Hospital. East Afr Med J.

[15] Binka C, Nyarko SH, Awusabo-Asare K, Doku DT (2018). "I always tried to forget about the condition and pretend I was healed": coping with cervical cancer in rural Ghana. BMC Palliat Care.

[16] Wuest J (2000). Repatterning care: women's proactive management of family caregiving demands. Health Care Women Int.

[17] Meleis AI, Lindgren TG (2002). Man works from sun to sun, but woman's work is never done: insights on research and policy. Health Care Women Int.

[18] Glazer NY (1990). THE HOME AS WORKSHOP: Women as Amateur Nurses and Medical Care Providers. Gend Soc.

[19] Gottschlich A, Rivera-Andrade A, Grajeda E, Alvarez C, Mendoza Montano C, Meza R (2017). Acceptability of human papillomavirus self-sampling for cervical cancer screening in an indigenous community in Guatemala. J Glob Oncol.

[20] Cancer Research UK KsCLaUCL: Breast module of the Cancer Awareness Measure (Breast-CAM): Toolkit. 2011.

[21] R Core Team (2021). R: A language and environment for statistical computing.

[22] Lund L, Ross L, Petersen MA, Groenvold M (2014). Cancer caregiving tasks and consequences and their associations with caregiver status and the caregiver’s relationship to the patient: a survey. BMC Cancer.

[23] Kurtz ME, Kurtz JC, Given CW, Given B (1995). Relationship of caregiver reactions and depression to cancer patients' symptoms, functional states and depression—A longitudinal view. Soc Sci Med.

[24] Hamid W, Khan TA (2021). Experiences of social support among Kashmiri women with breast cancer. Health Risk Soc.

[25] Fisher CL (2010). Coping with breast cancer across adulthood: emotional support communication in the mother-daughter bond. J Appl Commun Res.

[26] Raveis VH, Karus DG, Siegel K (1998). Correlates of depressive symptomatology among adult daughter caregivers of a parent with cancer. Cancer Interdiscip Int J Am Cancer Soc.

[27] Shrestha G, Mulmi R, Phuyal P, Thakur RK, Siwakoti B (2020). Experiences of cervical cancer survivors in Chitwan, Nepal: A qualitative study. PLoS ONE.

[28] Obi SN, Ozumba BC (2008). Cervical cancer: socioeconomic implications of management in a developing nation. J Obstetr Gynaecol.

[29] Noor-Mahomed SB, Schlebusch L, Bosch BA (2003). Suicidal behavior in patients diagnosed with cancer of the cervix. Crisis J Crisis Intervent Suicide Prev.

[30] Rosser JI, Zakaras JM, Hamisi S, Huchko MJ (2014). Men’s knowledge and attitudes about cervical cancer screening in Kenya. BMC Womens Health.

[31] Binka C, Doku DT, Awusabo-Asare K (2017). Experiences of cervical cancer patients in rural Ghana: An exploratory study. PLoS ONE.

[32] Maree JE, Mosalo A, Wright SCD (2013). 'It depends on how the relationship was before you became ill': black South African women's experiences of life partner support through the trajectory of cervical cancer. Eur J Cancer Care.

[33] Wilson TD (2014). Violence against Women in Latin America. Lat Am Perspect.

[34] Driscoll SD, Goodman R (2019). Emancipatory Praxis for Cervical Cancer Health Equity in Guatemala. Int J Human Caring.

[35] Waller J, Marlow LAV, Wardle J (2007). The association between knowledge of HPV and feelings of stigma, shame and anxiety. Sexually Transmitted Infections.

[36] Mendez-Luck CA, Kennedy DP, Wallace SP (2009). Guardians of health: The dimensions of elder caregiving among women in a Mexico City neighborhood. Soc Sci Med.

[37] Spitze G, Logan J. Sons, daughters, and intergenerational social support. J Marriage Fam 1990;52(2):420–30.

[38] Nguyen H, Nguyen T, Tran D, Hinton L (2021). “It’s extremely hard but it’s not a burden”: A qualitative study of family caregiving for people living with dementia in Vietnam. PLoS ONE.

[39] Perrin PB, Panyavin I, MorlettParedes A, Aguayo A, Macias MA, Rabago B, Picot SJF, Arango-Lasprilla JC (2015). A Disproportionate Burden of Care: Gender Differences in Mental Health, Health-Related Quality of Life, and Social Support in Mexican Multiple Sclerosis Caregivers. Behav Neurol.

[40] Ferrant G, Pesando LM, Nowacka K (2014). Unpaid Care Work: The missing link in the analysis of gender gaps in labour outcomes.

[41] Arrossi S, Matos E, Zengarini N, Roth B, Sankaranayananan R, Parkin M (2007). The socio-economic impact of cervical cancer on patients and their families in Argentina, and its influence on radiotherapy compliance. Results from a cross-sectional study. Gynecol Oncol.

[42] Hailu A, Mariam DH (2013). Patient side cost and its predictors for cervical cancer in Ethiopia: a cross sectional hospital based study. BMC Cancer.

[43] Tadesse SK (2015). Socio-economic and cultural vulnerabilities to cervical cancer and challenges faced by patients attending care at Tikur Anbessa Hospital: a cross sectional and qualitative study. BMC Womens Health.

[44] Gender Data Portal: Guatemala [https://genderdata.worldbank.org/countries/guatemala]. Accessed 28 Mar 2023.

[45] Sachs J, Kroll C, Lafortune G, Fuller G, Woelm F: Sustainable development report 2022. Cambridge: Cambridge University Press; 2022.

